# Gemcitabine resistance by CITED4 upregulation via the regulation of BIRC2 expression in pancreatic cancer

**DOI:** 10.1186/s12929-025-01140-y

**Published:** 2025-05-19

**Authors:** Eun-Jeong Jeong, Yuna Roh, Eunsun Jung, Jin-Seong Hwang, Taesang Son, Hyun Seung Ban, Tae-Su Han, Young-Kug Choo, Jang-Seong Kim

**Affiliations:** 1 Biotherapeutics Translational Research Center, Division of Biomedical Science, Korea Research Institute of Bioscience and Biotechnology, 125 Gwahak-Ro, Yuseong-Gu, Daejeon, 34141 Republic of Korea; 2 University of Science and Technology (UST), Daejeon, 34141 Republic of Korea; 3 Department of Biological Science, College of Health Sciences, Wonkwang University, Iksan, 54538 Republic of Korea; 4 School of Medicine, Sungkyunkwan University, Suwon, 16419 Republic of Korea; 5 Department of Otorhinolaryngology, College of Medicine, Konyang University Hospital, Konyang University Myunggok Medical Research Institute, Daejeon, 35365 Republic of Korea

**Keywords:** CITED4, BIRC2, Gemcitabine, Pancreatic cancer, Drug resistance

## Abstract

**Background:**

Gemcitabine (GEM) is used as a first-line therapy for patients diagnosed with any stage of pancreatic cancer (PC); however, patient survival is poor because of GEM resistance. Thus, new approaches to overcome GEM resistance in PC are urgently needed. Here, we aimed to establish an in vivo drug-resistant PC model and identify genes involved in GEM resistance. We focused on one of these factors, CITED4, and elucidated its mechanisms of action in GEM resistance in PC.

**Methods:**

L3.6pl, a GEM-sensitive PC cell line, was orthotopically injected into the pancreas of BALB/c nude mice to establish a GEM-resistant PC animal model. Transcriptomic data from control or GEM-resistant tumor-derived cells were analyzed. GEM resistance was evaluated using cell viability, clonogenicity, and apoptosis assays. An apoptosis array was used to identify genes downstream of *CITED4*. A *CITED4* knockout-mediated GEM sensitivity assay was performed in an orthotopic xenograft mouse model using PANC-1 cells, which are GEM-resistant cells.

**Results:**

From the RNA sequencing data of isolated GEM-resistant PC cells and The Cancer Genome Atlas dataset, 15 GEM resistance-related genes were found to be upregulated, including *CITED4*, the gene encoding a type of CBP/p300-interacting transactivator implicated in several cancers. *CITED4* knockdown in drug-resistant cells reduced cell proliferation and migration but increased apoptosis. To identify the molecular mechanism underlying CITED4-mediated induction of GEM resistance, alterations in Baculoviral IAP Repeat Containing 2 (BIRC2) levels were observed using an apoptosis array. *BIRC2* expression was downregulated following *CITED4* knockdown in GEM-resistant PC cell lines. Furthermore, chromatin immunoprecipitation and promoter assays showed that *BIRC2* was directly regulated by *CITED4*. Consistent with the *CITED*-knockdown experiments, silencing of *BIRC2* increased the sensitivity of L3.6pl-GEM-resistant and PANC-1 cell lines to GEM. Furthermore, *CITED4* knockout using the CRISPR-Cas9 system in PANC-1 cells increased the sensitivity to GEM in orthotopic mice. Moreover, elevated CITED4 and BIRC2 expression levels were associated with poorer outcomes in human PC clinical samples.

**Conclusions:**

Collectively, these results indicate that CITED4 regulates GEM resistance via inhibition of apoptosis by upregulating BIRC2 expression in PC cells. Therefore, CITED4 may serve as a valuable diagnostic marker and therapeutic target for GEM-resistant PC.

**Supplementary Information:**

The online version contains supplementary material available at 10.1186/s12929-025-01140-y.

## Introduction

Pancreatic cancer (PC) is an extremely aggressive cancer and is the third leading cause of cancer-related deaths in the United States [1–4]. In 2023, approximately 50,550 deaths due to PC and more than 64,050 new PC cases were expected to be reported in the USA [5, 6]. Most patients with PC are diagnosed at an advanced stage, and approximately 50% of patients with PC already have metastatic disease, because of the difficulty in distinguishing between chronic pancreatitis and benign pancreatic cysts. Thus, only less than 10% of patients with PC survive up to 5 years following diagnosis [7–12]. Variable clinical strategies are available for the management of PC, including surgical interventions, as well as adjuvant and neoadjuvant treatments. The first-line drug for PC treatment is gemcitabine (GEM), which inhibits DNA synthesis through the reduction of ribonuclease reductase activity, resulting in the activation of the apoptotic pathway, ultimately leading to cell death [13]. Nevertheless, a significant portion of patients fail to achieve a cure with GEM, primarily due to the development of chemoresistance during continuous GEM administration.

GEM-resistance mechanisms can alter GEM metabolism, decrease intracellular drug accumulation, inhibit apoptotic pathways, and cause abnormal activity of various signals that modulate the cell cycle and apoptosis. Proteins related to GEM metabolism pathways, including hENT1 and the rate-limiting enzyme dCK, are dysregulated, and RRM1/RRM2 is upregulated [14, 15]. High expression levels of Hu antigen R (HuR) are correlated with improved survival in GEM-treated patients, indicating that HuR is also associated with GEM efficacy [16]. Another mechanism of GEM resistance involves high expression of drug efflux pumps of the ABC transporter family [17]. These proteins are frequently expressed in cancer stem cells and protect against chemotherapeutic agents [18]. Additionally, several signaling pathways, such as the hedgehog, WNT, and notch signaling pathways, are reactivated in GEM-resistant cancer cells [19–21]. Although the mechanisms underlying GEM resistance in PC have been elucidated, resistance remains a major impediment to achieving satisfactory clinical outcomes thus, the identification of new therapeutic targets is needed to overcome GEM resistance.

CBP/p300-Interacting Transactivator with ED-rich Tail 4 (CITED4) is a transcriptional co-regulator involved in various cellular processes. Recent studies have suggested that CITED4 plays a crucial role in cancer development and progression. In lung cancer, it drives cell proliferation through the HB-EGF–STAT3–MYC pathway [22]. In colorectal cancer, CITED4 silencing leads to G2 cell cycle arrest and disrupts adhesion-related gene expression, affecting tumor invasion [23]. In breast cancer, its cytoplasmic localization increases HIF-1α expression, promoting tumor aggressiveness. Beyond its role in tumor growth, CITED4 is also a potential biomarker [24]. In lung adenocarcinoma, it enhances metastasis by upregulating CLDN3 through the Wnt/β-catenin pathway [25]. These findings indicate that CITED4 is a key regulator of cancer proliferation, invasion, metastasis, and therapeutic response. Further research is needed to explore its potential as a therapeutic target and a biomarker for early cancer detection and treatment.

In this study, our primary objective was to investigate the functions of CITED4 and elucidate the novel downstream signaling pathway in GEM-resistant PC and its underlying mechanism, specifically focusing on the role of CITED4 in apoptosis. Notably, silencing of CITED4 or BIRC2 led to the inhibition of cell proliferation and an increase in cellular apoptosis. Further, CITED4 knockout (KO) in a highly GEM-resistant cell, PANC-1, significantly suppressed the growth of orthotopically injected tumor cells following GEM treatment, indicating that CITED4 is a potential key regulator of GEM resistance in PC.

## Methods

### Cell culture

Human PC cell line L3.6pl was a kind gift of Dr. Sun Jin Kim (The University of Texas M.D. Anderson Cancer Center, Houston, Texas, USA). L3.6pl, a GEM-sensitive cell line, was maintained in Dulbecco’s modified Eagle medium (DMEM, Welgene, Gyeongsan-si, Korea) supplemented with 10% fetal bovine serum (FBS, RMBIO, Missoula, MT, USA), antibiotic–antimycotic (Thermo Fisher Scientific, Waltham, MA, USA), nonessential amino acids (Thermo Fisher Scientific), and 2 × MEM vitamin solution (Thermo Fisher Scientific). PANC-1, a GEM-resistant cell line, was purchased from the Korean Cell Line Bank (#21469; Seoul, Korea). The PANC-1 cell line was also maintained in DMEM supplemented with 10% FBS and antibiotic–antimycotic. To generate luciferase-expressing cells, L3.6pl and PANC-1 cells were pre-incubated for 1 h with 8 μg/ml polybrene (Sigma-Aldrich, St. Louis, MO, USA). Then, lentiviral particles (Capital Biosciences, Gaithersburg, MD, USA) containing a luciferase gene were transduced into the cells. After transduction, the cells were treated with 15 μg/ml puromycin for selection of luciferase-positive cells (Thermo Fisher Scientific). For analyzing the effect of epigenetic modification by chemicals, the 0.2 μM trichostatin A (TSA, Sigma-Aldrich) and/or 5 μM 5-Aza-2′-deoxycytidine (5-Aza-dC, Sigma-Aldrich) were treated in the L3.6pl cells.

### Animal experiments

To establish a GEM-resistant mouse model, 1 × 10^6^ L3.6pl-luc cells were orthotopically injected into the pancreas of BALB/c-nu female mice (Harlan Laboratories, Indianapolis, IN, USA). The mice were maintained in a controlled environment at a temperature of 23 ± 1 ℃, humidity of 55 ± 5%, and noise levels below 70 dB, with a 12 h photoperiod (lights were switched on at 6 AM and switched off at 6 PM). After 2 weeks, phosphate-buffered saline (PBS, control) or GEM (50 mg/kg) was intraperitoneally administered to each mouse, twice a week, for 9 weeks. Bioluminescent images of mice were obtained every week using an In Vivo Imaging System 200 (IVIS200, Perkin Elmer, Waltham, MA, USA), and photon counts were analyzed using the Living Image® software 4.0 (Caliper Life Sciences, Hopkinton, MA, USA). The mice were analyzed at 6–9 weeks, depending on the tumor size, and the tumor was removed and used to establish primary cell cultures. We named these cell lines derived from PBS- or GEM-treated tumor cells as Ctrl-L3.6pl and GEM-exposed L3.6pl (GE-L3.6pl), respectively. To evaluate GEM resistance of CITED4-knockout (KO) PANC-1 cells, we divided them into three groups: PANC-1 cells as the control group, PANC-1-CITED4-CAS9-#1 as the CITED4-KO-#1 group, and PANC-1-CITED4-CAS9-#3 as the CITED4-KO-#3 group. Cells (1 × 10^6^) were orthotopically injected into the pancreas of BALB/c-nu mice (Orient Bio., Seongnam, Korea), and PBS or GEM (50 mg/kg) was administered intraperitoneally to each mouse, twice a week, for 7 weeks. Quantitative analysis of tumor size was performed via bioluminescent imaging using an IVIS200 instrument, Regions of interest from captured images were analyzed based on the tumor sites and quantified as total photon counts with Living Image^®^ software (PerkinElmer) [26]. The body weight was measured weekly. After 7 weeks, tumors were isolated from mice, and the tumor weights were measured. All animal experiments were approved by the Committee on Animal Experimentation of the Korea Research Institute of Bioscience and Biotechnology.

### Patient samples

In this study, we collected normal pancreatic tissues (*n* = 11) and pancreatic cancer tissues (*n* = 25) from CHA University Bundang Medical Center, a member of the Korea Biobank Network. This study was approved by the Public Institutional Review Board of the Ministry of Health and Welfare (P01-202105-31-011).

### RNA extraction and quantitative reverse transcription-PCR

Total RNA was extracted from PC cell lines and tumor tissues using Nucleozol (Macherey–Nagel GmbH & Co., KG, Düren, Germany) following the manufacturer’s instructions. cDNA was synthesized with PrimeScript™ RT reagent kit (Takara, Osaka, Japan), following the manufacturer’s instructions. Quantitative real-time PCR (qPCR) was performed with the Step One Plus real-time PCR system (Applied Biosystems, Waltham, MA, USA) using Fast SYBR Green Master Mix (Applied Biosystems). The expression levels of *GAPDH* and *ACTB* mRNAs were used to normalize the expression level of mRNA. The sequences of primers used in this study are listed in Table S1.

### Small interfering RNA

PANC-1 or L3.6pl cells were transfected with a negative control (siNC), CITED4 (siCITED4), or BIRC2 (siBIRC2) small interfering RNA (Bioneer, Daejeon, Korea) using the RNAiMAX transfection reagent (Thermo Fisher Scientific) following the manufacturer’s instructions. The siRNA sequences are listed in Table S2.

### CRISPR/Cas9-CITED4 gene editing

For the construction of CITED4-KO cells, the CRISPR/Cas9 gene editing system was used. Briefly, sgRNA sequences (Table S2) were inserted into the pSpCAS9-2 APuro vector, and the sequences were verified. The CITED4-KO vector was transfected into PANC-1 cells using Lipofectamine^®^ 2000 Transfection Reagent (Thermo Fisher Scientific) for 24 h, and the transfected cells were selected with puromycin (15 μg/ml).

### Assays of cell proliferation, migration, and clonogenicity

Cell proliferation and viability were measured using a Cell Counting Kit 8 (CCK-8) assay (Dojindo, Kumamoto, Japan). PANC-1 cells (1 × 10^3^ cells/well) or L3.6pl (3 × 10^3^ cells/well) were seeded into 96-well plates. After 24 h, GEM was added to each well, and the plate was incubated for 48 h. Then, 10 μl of CCK-8 was added into each well and the cell medium were measured at 450 nm using a microplate reader (Tecan, Männedorf, Switzerland).

For the cell migration assay, the PC cells were seeded in the upper compartment of a transwell chamber (Corning Inc., New York, NY, USA) in 200 μl of serum-free medium, and 700 μl of complement culture medium was added to the lower compartment. After 48–72 h, the cells remaining on the upper membrane were removed with cotton wool. The cells were fixed with methanol and then stained with 0.1% crystal violet (Sigma-Aldrich).

For the clonogenicity assay, PANC-1 and L3.6pl cells were seeded in 6-well plates at a density of 500 cells/well. After 21 days, the cells were fixed with methanol and then stained with 0.1% crystal violet (0.1% w/v, Sigma-Aldrich). Visible colonies were counted under an inverted microscope.

### Western blotting

AsPC-1, PANC-1, or L3.6pl cells were lysed in RIPA buffer (Thermo Fisher Scientific) containing EDTA solution (Thermo Fisher Scientific) and phosphatase and protease inhibitors (Thermo Fisher Scientific). The lysates were incubated on ice for 30 min and centrifuged at 12,000 × g for another 30 min at 4 °C. The protein concentration was measured using a bicinchoninic acid assay kit (Thermo Fisher Scientific). Equal amounts of total protein (30 μg) from each cell line were separated using Mini-PROTEAN TGX gels (Bio-Rad, Hercules, CA, USA) and electrotransferred to polyvinylidene difluoride membranes (Bio-Rad) using the Trans-Blot Turbo Transfer System (Bio-Rad). The membranes were blocked with 5% bovine serum albumin (BSA) at 25 °C for 1 h and then incubated with a primary antibody overnight at 4 ℃. The membranes were washed with PBS containing 0.1% Tween-20 and then probed with horseradish peroxidase (HRP)-conjugated secondary antibodies. Protein bands were detected using ECL Prime Western Blotting Detection Reagent (GE Healthcare, Chicago, IL, USA) following the manufacturer’s instructions. The antibodies used in the experiments are listed in Table S3.

### Apoptosis assay

L3.6pl-GEM resistant (GR) or PANC-1 cells were grown in complete culture media in the presence of GEM (1000 ng/ml) for 48 h. The cells were then washed with PBS twice, and cell pellets were resuspended in 1 × binding buffer to a concentration of 1 × 10^5^ cells/100 μl media. The cells were incubated with 5 μl of Annexin V-FITC and propidium iodide (PI; BD Biosciences) solutions in the dark. After 15 min, 400 μl of 1 × binding buffer was added to each tube, and the samples were analyzed using FACS Verse (BD Biosciences).

### Apoptosis array

Apoptosis-related protein expression profiling was performed using a human apoptosis array (R&D Systems, Minneapolis, MN, USA) following the manufacturer’s protocol. Then, 2 × 10^6^ of L3.6pl-GR cells were seeded and transfected with siNC or siCITED4. The transfected cells were harvested, lysed using a lysis buffer, and quantified using the Pierce BCA Protein Assay Kit (Thermo Fisher Scientific). The membranes were blocked with 5% BSA at 25 °C for 1 h and then incubated with cell lysates overnight at 4 ℃. The membranes were washed with PBS containing 0.1% Tween-20 and then probed with HRP-conjugated secondary antibodies. Protein bands were detected using ECL Prime Western Blotting Detection Reagent (GE Healthcare) following the manufacturer’s instructions. Images were quantified using ImageJ software (NIH, Bethesda, MD, USA).

### Immunostaining

For the human tissue microarray, paraffin-embedded glass slides, including 20 PC and 4 normal and non-malignant pancreatic tissue samples, were purchased from Biochain (Newark, CA, USA; cat. Z7020090), and CITED4 staining was performed by immunohistochemistry (IHC). For IHC, the sections were deparaffinized in xylene prior to rehydration using an alcohol gradient. Endogenous peroxidase activity was blocked using 3% hydrogen peroxide in PBS for 20 min. For antigen retrieval, the sections were treated with solution A + B buffer (citric acid + trisodium) for 15 min at 95 °C in a microwave oven. After blocking with 2.5% normal goat serum for 1 h at 25 °C, the sections were incubated with primary antibodies overnight at 4 °C. The primary antibodies used were as follows: rabbit monoclonal anti-CITED4 (Abcam, Cambridge, MA, USA; dilution 1:100), rabbit monoclonal anti-BIRC2 (Abcam, dilution 1:100), and rabbit polyclonal anti-cleaved Caspase-3 (Cell Signaling, dilution 1:100). Following incubation, the sections were washed with PBS and incubated with HRP-conjugated goat anti-rabbit secondary antibody for 30 min at 25 °C. Staining was performed using 3,3′-diaminobenzidine (DAB; Vector Laboratories, Burlingame, CA, USA). The sections were counterstained with hematoxylin, dehydrated, and mounted. The antibodies used in this study are listed in Table S3.

For immunocytochemistry, cells were fixed with 4% paraformaldehyde for 15 min at 25 °C, permeabilized with 0.1% Triton X-100 for 15 min, and blocked with 4% BSA (Sigma-Aldrich) for 1 h at 25 °C. The samples were stained with the respective primary antibodies diluted in blocking buffer overnight at 4 °C, washed with 0.05% Tween-20 (Sigma-Aldrich) in PBS, and then incubated with Alexa Fluor-conjugated secondary antibodies (Thermo Fisher Scientific) for 1 h at 25 °C. Finally, the cells were mounted using a mounting solution containing DAPI (Thermo Fisher Scientific). Fluorescent images were obtained using a confocal microscope (Carl Zeiss, Oberkochen, Germany). The antibodies used in the experiments are listed in Table S3.

### Chromatin immunoprecipitation assay

The chromatin immunoprecipitation (ChIP) assay was performed as previously described [27]. The CITED4-binding motif in the promoter region of *BIRC2* was identified using JASPAR software (http://jaspar.genereg.net/). The promoter region sequences were confirmed using the Eukaryotic Promoter Database (EPD; https://epd.epfl.ch/). ChIP was performed using Dynabeads Protein A and G protocols (Thermo Fisher Scientific). The antibodies used for ChIP are listed in Table S3. Immunoprecipitated DNA and input samples were analyzed using real-time qPCR with specific primers listed in Table S1.

### Luciferase reporter assays

For the promoter assay, the TFAP2 A-binding sequences (pGL4-TFAP2 A-var3 [E1]) in the *CITED4* promoter site were cloned into a pGL4 luciferase reporter vector (Promega Corp., Madison, WI, USA). The Tk-*Renilla* plasmid DNA, pGL4-TFAP2 A-var3 [E1] plasmid DNA and 50 nM siNC or siCITED4 were co-transfected into PANC-1 cells using Lipofectamine 2000 (Thermo Fisher Scientific). After 48 h, the cells were harvested to analyze luciferase activity using a dual-luciferase reporter assay system (Promega). Luciferase activity was determined as the ratio of firefly luciferase activity to *Renilla* luciferase activity.

### RNA sequencing and data analysis

In brief, total RNA was extracted, and an oligo-dT primer containing an Illumina-compatible sequence at the 5’ end was annealed to the RNA template, initiating reverse transcription. After the RNA template was degraded, second-strand synthesis was performed using a random primer with an Illumina-compatible linker at the 5’ end. The resulting double-stranded cDNA was then purified with magnetic beads, ensuring the removal of all reaction components. Subsequently, the library underwent PCR amplification to incorporate the complete adapter sequences required for cluster generation, followed by an additional purification step to remove any residual PCR components. The final library was subjected to high-throughput single-end sequencing (75 bp) on the NextSeq 500/550 platform (Illumina Inc.). Data mining and graphic visualization were performed using ExDEGA (Ebiogen Inc., Korea). For functional annotation and pathway enrichment analysis, Gene Ontology (GO) and Kyoto Encyclopedia of Genes and Genomes (KEGG) pathway analyses were conducted using DAVID (Database for Annotation, Visualization, and Integrated Discovery) version 6.8. and GSEA (Gene Set Enrichiment Analysis).

### Data availability

Public datasets on survival in patients with PC were obtained from the Q-omics software [28]. The survival curves were generated using the Kaplan–Meier method and were compared using the log-rank test. The correlation between gene expression in PC patients was obtained from the Gene Expression Profiling Interactive Analysis 2 (GEPIA2, http://gepia2.cancer-pku.cn/) including PAAD normal, tumor, and GTEx normal pancreas datasets [29]. The Cancer Genome Atlas (TCGA)-pancreatic adenocarcinoma (PAAD) was analyzed, and clinical information and transcriptome data of TCGA samples were downloaded from the University of California-Santa Cruz Cancer Browser (https://xena.ucsc.edu/). The patients’ clinical data and status of PC primary therapy outcome success were obtained from TCGA-PAAD database.

To investigate the relationship between DNA methylation and CITED4 expression, we utilized publicly available datasets and bioinformatics tools. Specifically, we analyzed the methylation status of the CITED4 promoter region using MEXPRESS and the Shiny Methylation Analysis Resource Tool (SMART) [30, 31]. MEXPRESS (https://mexpress.be) was used to visualize and evaluate the correlation between CpG site methylation and CITED4 expression levels in PC samples. Additionally, we employed SMART (https://shiny-methylation.com) to further validate methylation patterns and assess their potential association with clinical outcomes, including patient prognosis. For each analysis, CpG site methylation values were extracted and statistically correlated with CITED4 expression levels using Spearman’s correlation coefficient, suggesting a potential clinical significance of CITED4 epigenetic regulation in pancreatic cancer.

### Statistical analysis

All statistical data were analyzed using GraphPad Prism, version 10.4 (GraphPad Inc., La Jolla, CA, USA). All data are presented as mean ± standard deviation, unless otherwise indicated. Depending on the sample size, the Kolmogorov–Smirnov test (*n* > 50) or Shapiro–Wilk test (*n* < 50) was performed to test normality of data distribution. The unpaired Student’s *t*-test or Mann–Whitney U test was used to analyze differences between the two groups. For the comparison of multiple groups, one-way analysis of variance (ANOVA) was performed along with post-hoc multiple comparison tests, including the Student–Newman–Keuls, Dunnett’s, and Tukey’s tests. Pearson correlation analysis was used for correlation analysis of expression of each gene. Survival was analyzed using the Kaplan–Meier method and log-rank test. *P* < 0.05 was considered to indicate statistical significance.

## Results

### Establishment of the GEM-resistant cell line and NGS-based discovery of CITED4

**Fig. 1:**
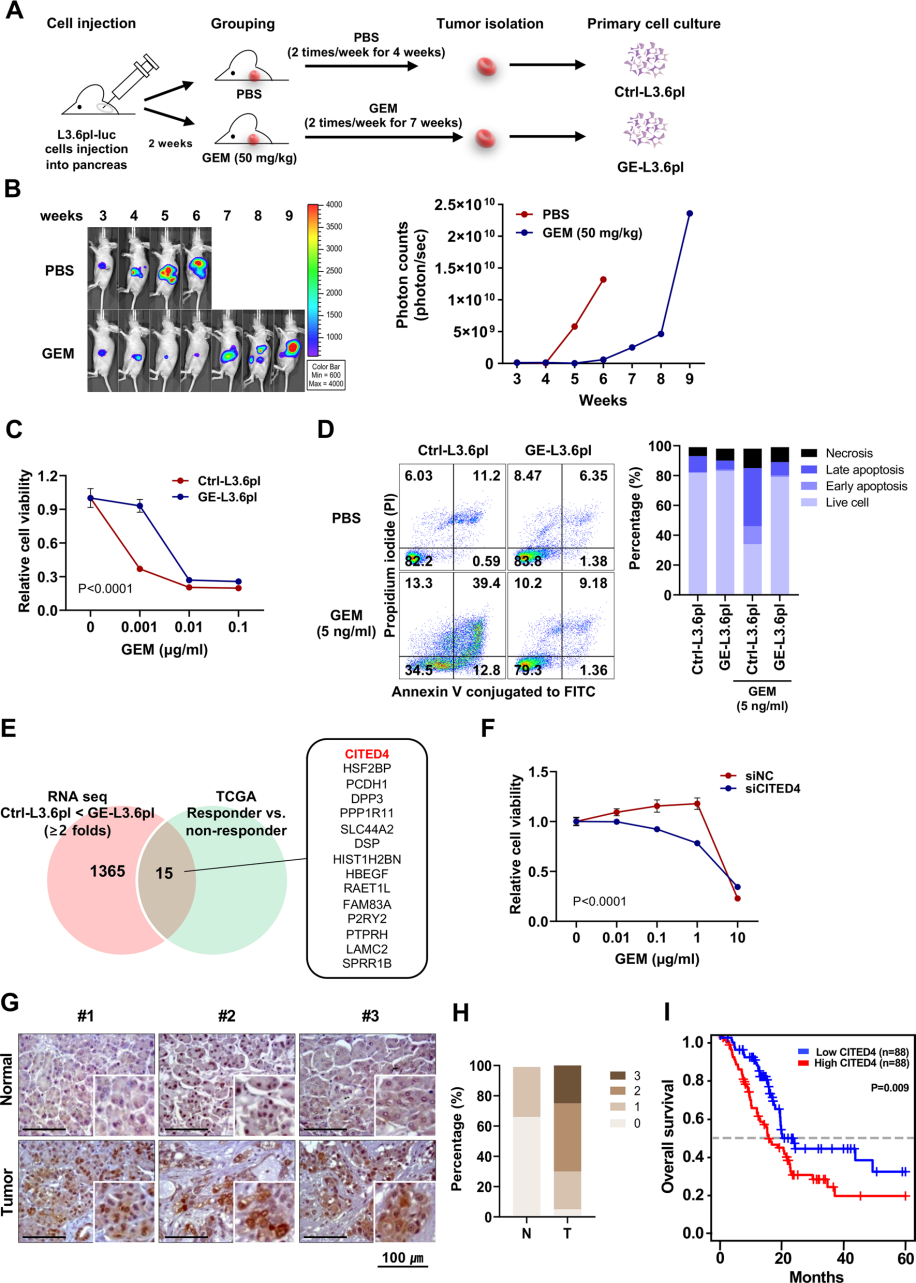
Identification of gemcitabine (GEM)-resistance genes in PC.** A** Schematic representation of the preparation of a GEM-resistant PC cell line. **B** Mice were subjected to bioluminescent imaging using an in vivo imaging system. Bioluminescent images were captured once a week post-cell injection, and representative images are shown (left panel). The levels of bioluminescent intensity (total photon flux per second) in the pancreatic regions were quantified and compared between the PBS and GEM treatment groups (right panel). (PBS, *n* = 6; GEM, *n* = 6). **C** Cell viability was measured using a CCK-8 assay. Control cells (GEM-sensitive pancreatic cells from PBS-injected mice) and GE-L3.6pl cells (GEM-resistant pancreatic cells from GEM-injected mice) were seeded at a density of 3 × 10^3^ cells/well in 96-well plates, and treated with various concentrations of GEM for 48 h. **D** Fluorescence-activated cell sorting analysis of apoptosis. Representative flow cytometry dot plots with double Annexin V-FITC/propidium iodide staining for control and GE-L3.6pl cells exposed to GEM (5 ng/ml) for 48 h (left panel). Percentages of viable (white bars), early apoptotic (light gray bars), late apoptotic (dark gray bars), and necrotic (black bar; right panel) cells. **E** Venn diagram showing 15 genes correlated with GEM resistance between The Cancer Genome Atlas data and RNA sequencing analysis. **F** Cell viability was measured using a CCK-8 assay after cells were transfected with siCITED4 and treated with various concentrations of GEM for 48 h. **G** Immunohistochemical staining of CITED4 in tumorous and non-tumorous pancreatic tissues (scale bar, 100 μm). **H** Expression pattern of CITED4 in the human pancreatic tumor tissue array. Diagnostic score 0 (beige), score 1 (light brown), score 2 (brown), and score 3 (dark brown). **I** Overall survival analysis for PC samples from Q-omics (https://qomics.sookmyung.ac.kr/) depending on CITED4 expression

In vivo chemoresistance models have several advantages owing to their heterogeneous nature and the use of a native microenvironment, which includes inflammation [32]. Thus, we generated an in vivo mouse model of GEM-resistant PC to discover novel targets associated with GEM resistance in PC. To establish a GEM-resistant PC model, GEM-sensitive L3.6pl-luc PC cells were orthotopically injected into the pancreas of BALB/c nude mice, and PBS or GEM was intraperitoneally administered for 9 weeks (Fig. 1A). As shown in Fig. 1B, luciferase activity gradually increased after 4 weeks in the PBS-treated group. In contrast, GEM-treated mice showed suppressed tumor growth until 6 weeks; however, after 7 weeks, the tumor size eventually increased owing to GEM resistance. Next, we cultured primary cells from the PBS (Ctrl-L3.6pl) and GEM-exposed (GE-L3.6pl) tumors. Short tandem repeat analysis revealed that the primary cultured cells matched the L3.6pl parental cells (Supplementary Table S4). Next, we performed cell viability and apoptosis assays were performed to determine the drug resistance of the GE-L3.6pl cells. After treatment with different concentrations of GEM, the cell viability significantly increased in GE-L3.6pl cells as compared to that in Ctrl-L3.6pl cells (Fig. 1C). Moreover, flow cytometric analysis showed that the number of apoptotic cells increased in GEM-treated Ctrl-L3.6pl cells (52.2%) as compared with that in GE-L3.6pl cells (10.5%) (Fig. 1D).

Next, to identify novel targets associated with GEM resistance in PC, we conducted RNA sequencing of Ctrl-L3.6pl and GE-L3.6pl cells. We analyzed the transcriptome in relation to the primary therapy outcome success status from the PAAD clinical matrix of the TCGA dataset. We identified 15 upregulated genes using an integrated analysis of RNA sequencing and TCGA datasets (Fig. 1E). Among these 15 candidate genes, *CITED4* knockdown significantly increased the sensitivity to GEM in GE-L3.6pl cells (Fig. 1F). We further extended this investigation to clinical samples, in which CITED4 expression was examined using a tissue array of PC and non-malignant pancreatic tissues. Our findings revealed an upregulation of CITED4 expression in PC tissues relative to that in normal pancreatic tissues (Fig. 1G, H). Additionally, survival analysis using the Q-omics software showed a significant reduction in overall survival among PC patients who exhibited high CITED expression as compared to that in PC patients with low CITED4 expression (Fig. 1I), indicating that CITED4 expression is associated with GEM resistance and poor prognosis.

### CITED4 expression is upregulated in GEM-resistant cells

**Fig. 2:**
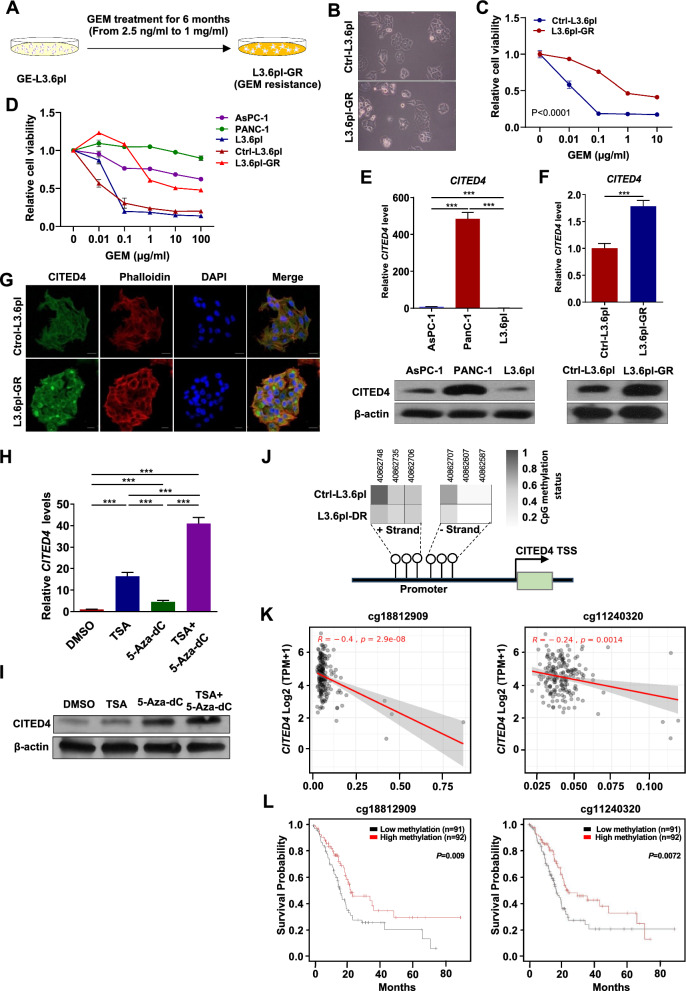
CITED4 expression is upregulated in GEM-resistant PC cells. **A** Schematic representation of stronger GEM resistance in cells (L3.6pl-GR). **B** Phase-contrast image for control (top) and L3.6pl-GR (bottom). **C** Cell viability was measured using a CCK-8 assay in control and L3.6pl-GR cells seeded at a density of 3 × 10^3^ cells/well in 96-well plates, and treated with various concentrations of GEM for 48 h. **D** Cell viability was measured using a CCK-8 assay. PANC-1 (green circle), AsPC-1 (purple circle), L3.6pl-GR (red triangle), control (brown triangle), and L3.6pl (blue triangle) cells were treated with various concentrations of GEM for 48 h. **E** *CITED4* mRNA and protein expression levels in AsPC-1, PANC-1, and L3.6pl PC cell lines. **F** *CITED4* mRNA and protein expression levels in Ctrl-L3.6pl and L3.6pl-GR cells. **G** Immunofluorescence staining of CITED4 (green), phalloidin (red), and DAPI (blue) was detected in control and L3.6pl-GR. Scale bar, 20 μm. **H** mRNA expression and **I** Protein expression after treatment of Trichostatin A (TSA, 0.2 μM) and/or DNA demethylation agent, 5-Aza-2’-deoxycytidine (5-Aza-dC, 5 μM) for 24 h. β-actin was used as an internal control for mRNA and protein expression. **J** DNA methylation status of CITED4 promoter region by DNA methylation amplicon sequencing for Ctrl-L3.6pl and L3.6pl-GR. **K** Scatter plots showing the correlation between CITED4 expression and DNA methylation levels at specific CpG sites (cg18812909, left; cg11240320, right). **L** Kaplan–Meier survival curves show the overall survival of PC patients depending on the DNA methylation status of CITED4 (cg18812909, left; cg11240320, right). ^***^P < 0.001

To maintain GEM resistance in GE-L3.6pl cells in vitro, the cells were continuously exposed to increasing GEM concentrations, from 2.5 ng/ml to 1 mg/ml, over 6 months (L3.6pl-GR, Fig. 2A). With an increase in GEM concentration, the cell viability decreased in control cells treated with GEM but not in L3.6pl-GR cells (Supplementary Fig. 1 A). Moreover, the Caspase3/7-Glo assay revealed that luciferase activity significantly increased in GEM-treated control cells as compared to that in PBS-treated cells, whereas no difference was observed between PBS- and GEM-treated L3.6pl-GR cells (Supplementary Fig. 1B). Although L3.6pl and L3.6pl-GR cells did not differ morphologically (Fig. 2B), GEM resistance significantly increased in L3.6pl-GR cells (Fig. 2C). Moreover, when compared with AsPC-1 and PANC-1 cells, which are well-known GEM-resistant cell lines, L3.6pl-GR cells had a similar drug response (Fig. 2D).

To determine whether CITED4 expression is associated with GEM resistance in various PC cell lines, qPCR and western blotting experiments were performed using AsPC-1, PANC-1, L3.6pl, and primary cultured cells from the in vivo mouse model, namely Ctrl-L3.6pl and L3.6pl-GR lines. We observed upregulated CITED4 RNA and protein expression in PANC-1 cells compared to that in AsPC-1 or L3.6pl cells (Fig. 2E). Moreover, the CITED4 level significantly increased in L3.6pl-GR cells as compared to that in Ctrl-L3.6pl cells (Fig. 2F). Consistent with these findings, immunofluorescence staining showed that the CITED4 level was elevated in L3.6pl-GR cells as compared to that in Ctrl-L3.6 pl cells (Fig. 2G).

Next, to determine whether CITED4 upregulation in GEM-resistant cells is associated with epigenetic regulation, we treated L3.6pl cells, which are CITED4-low expressing cells, with the deacetylase inhibitor Trichostatin A (TSA, 0.2 μM) and/or DNA demethylation agent, 5-Aza-2’-deoxycytidine (5-Aza-dC, 5 μM). TSA or 5-Aza-dC treatment significantly increased CITED4 expression. Furthermore, a combination treatment of TSA and 5-Aza significantly upregulated CITED4 mRNA and protein levels (Fig. 2H, I). To further investigate the relationship between DNA methylation at CITED4 promoter CpG sites and GEM resistance, we performed methylation bisulfite amplicon sequencing on Ctrl-L3.6pl and L3.6pl-GR cells. The results showed that CpG sites in L3.6pl-GR cells were hypomethylated compared to Ctrl-L3.6pl cells, which aligns with increased CITED4 expression and its potential role in GEM resistance (Fig. 2J). Additionally, we identified a correlation between CpG site methylation within the CITED4 promoter and its expression levels using public datasets (MEXPRESS and SMART). Specifically, two CpG sites (cg18812909 and cg11240320) showed an inverse correlation between DNA methylation and CITED4 expression (Fig. 2K). Furthermore, we found that hypomethylation of these CpG sites was associated with the prognosis of PC patients (Fig. 2L), suggesting a potential clinical significance of CITED4 epigenetic regulation in PC.

### Suppression of CITED4 decreases the oncogenic properties and induces cellular apoptosis in PC

**Fig. 3:**
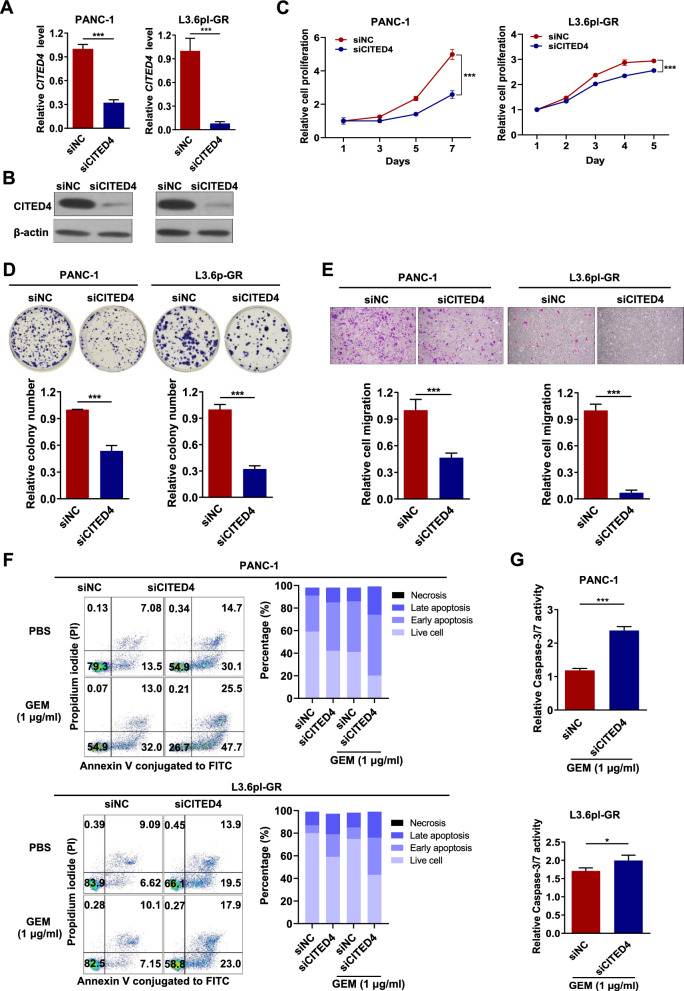
Oncogenic properties of CITED4 in PC. **A** *CITED4* mRNA expression level in *CITED4*-knockdown PANC-1 and L3.6pl-GR. *ACTB* was used as an internal control for mRNA expression analysis. **B** Protein expression level of CITED4 in *CITED4*-knockdown PANC-1 and L3.6pl-GR cells. β-actin was used as an internal control for mRNA and protein expression. **C** Cell viability was measured using a CCK-8 assay. *CITED4*-knockdown PANC-1 and L3.6pl-GR cells were cultured with 1 µg/ml of GEM for 7 days. **D** Representative image of the colony-formation assay for PANC-1 and L3.6pl-GR cells and relative quantification of the colony number. **E** Representative image of migration of PANC-1 and L3.6pl-GR cells and relative quantification of the number of migrated cells. **F** Comparative analysis of cellular apoptosis between siRNA for negative control (siNC) and siRNA for CITED4 (siCITED4) in PANC-1 (upper) and L3.6pl-GR (bottom) with GEM treatment using flow cytometry. FACS analysis of siNC- and siCITED4-transfected cells treated with or without GEM (1 µg/ml) for 48 h, following which the cells were stained with Annexin V-FITC and propidium iodide (left panel). Percentages of viable, early apoptotic, late apoptotic, and necrotic cells (right panel). **G** Caspase-3/7 activity was measured using a Caspase-Glo 3/7 Assay kit. *CITED4*-knockdown PANC-1(upper) and L3.6pl-GR (bottom) cells were treated with 1 µg/ml GEM for 48 h, following the manufacturer’s protocol. *P < 0.05 and ***P < 0.001

As CITED4 expression was upregulated in GEM-resistant PC cells, we investigated the cellular functions of CITED4 in a series of PC cell lines. We first knocked down CITED4 in PANC-1 and L3.6pl-GR cells using siCITED4. CITED4 knockdown in PANC-1 and L3.6pl-GR cells significantly reduced CITED4 mRNA (Fig. 3A) and protein (Fig. 3B) expression as compared with the negative control (siNC). Importantly, suppression of CITED4 expression significantly reduced the cell viability, clonogenicity, and migratory ability of PANC-1 and L3.6pl-GR cells (Fig. 3C–E).

Next, to investigate the apoptotic characterization of *CITED4* knockdown, siCITED4 was used to treat PANC-1 and L3.6pl-GR cells. Although siCITED4 treatment slightly increased the number of apoptotic cells, treatment with 1 μg/ml GEM knocked down CITED4 in PANC-1 and L3.6pl-GR cells for 48 h, resulting in an increased apoptosis rate as compared with siNC transfection (Fig. 3F). Additionally, siCITED4 significantly increased Caspase3/7-Glo activity in both PANC-1 and L3.6pl-GR cells as compared with siNC transfection following GEM treatment (Fig. 3G). Collectively, these findings indicate that CITED4 expression promotes the oncogenic properties and prevents GEM-induced cell death in PC via inhibition of cellular apoptosis.

### CITED4 expression is positively correlated with BIRC2 expression

**Fig. 4:**
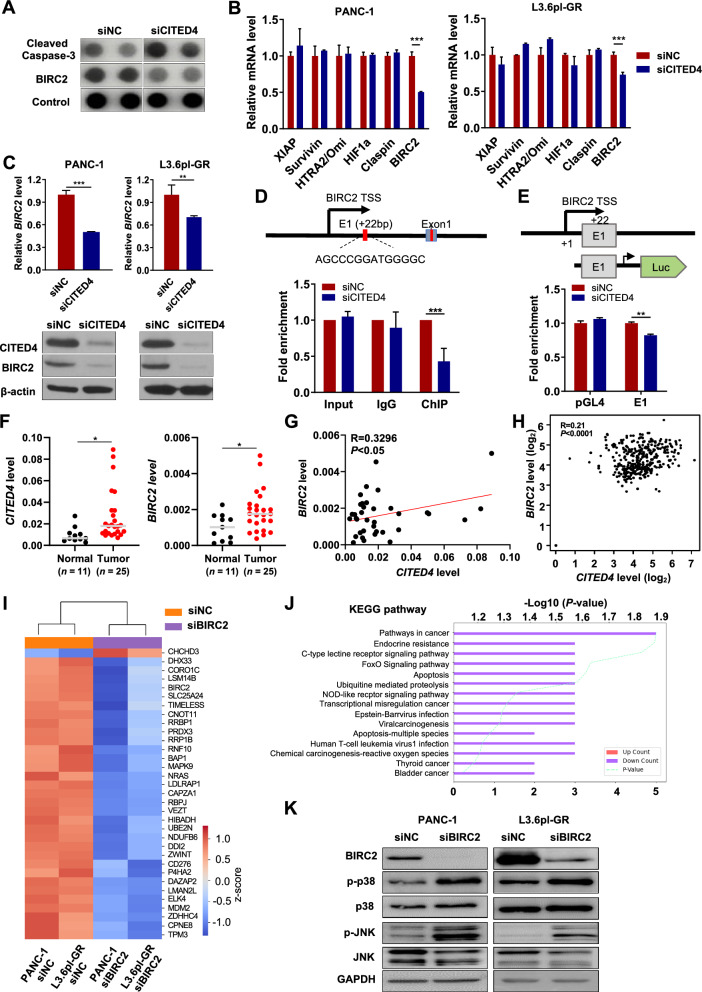
CITED4 is related to apoptosis and functions through BIRC2.** A** Proteome profiling of apoptosis-associated proteins. Array images showing cleaved Caspase-3 and BIRC2 expression in *CITED4*-knockdown L3.6pl-GR cells. **B** Apoptosis-related gene expression in CITED4-knockdown PANC-1 (left) and L3.6pl-GR (right) cells. **C** mRNA (top) and protein (bottom) expression levels of CITED4 in *CITED4*-knockdown PANC-1 and L3.6pl-GR cells. **D** Schematic representation of the *BIRC2* promoter region. Chromatin immunoprecipitation (ChIP)-qPCR was used to amplify chromatin derived from immunoprecipitation with CITED4 antibody, as indicated. **E** Luciferase reporter assay of *BIRC2* promoter activity and schematic representation of the truncated promoter plasmid (upper panel). Relative luciferase activity was determined using the ratio of firefly luciferase/*Renilla* luciferase activity (bottom panel). **F** *CITED4* and *BIRC2* expression levels in normal pancreatic tissues and PC tumor tissues. **G** Correlation analysis of between *CITED4* and *BIRC2* expression levels using tissue samples. **H** Correlation analysis of between *CITED4* and *BIRC2* expression levels from PAAD-TCGA using GEPIA2 (R = 0.21, *P* < 0.0001). **I** Heat map of the differentially expressed genes (DEGs) in *BIRC2*-knockdown PANC-1 and L3.6pl-GR. Representative genes are shown on the right. **J** The Database for Annotation, Visualization, and Integrated Discovery (DAVID) tool was used to perform KEGG pathway analysis for DEGs. **K** The expression of CITED4, BIRC2, phosphorylated p38 MAPK (p-p38), total p38 MAPK (p38), phosphorylated JNK (p-JNK), and total JNK in BIRC2 knockdown PACN-1 and L3.6pl-GR cells. GAPDH were used as an internal control for protein expression. **P < 0.01 and ***P < 0.001

As discussed previously, PC cells may possess strategies involving CITED4 expression to prevent cell death induced by GEM. To elucidate the effect of CITED4 on apoptosis and its potential role in increasing GEM resistance, an apoptosis assay was performed for siCITED4-treated PANC-1 cells. Silencing of *CITED4* in PANC-1 cells showed that various proteins were upregulated or downregulated according to their role in apoptotic pathways (Supplementary Fig. 2). Specifically, CITED4 knockdown increased the level of cleaved Caspase-3 and decreased the level of BIRC2 (Fig. 4A).

According to a previous report, CITED4 acts as a transcription factor cooperating with TFAP2 A [33]; thus, we analyzed the mRNA expression levels of candidate genes downstream of CITED4 using qPCR. We found that *CITED4* knockdown decreased the *BIRC2* mRNA level as well as the BIRC2 protein level in PANC-1 and L3.6pl-GR cells (Fig. 4B, C). To ascertain whether *BIRC2* levels are directly modulated by CITED4 as a transcription factor, we performed a chromatin immunoprecipitation (ChIP) assay. Using the Eukaryotic Promoter Database (EPD), we found that the TFAP2 A-binding site was located at + 22 bp from the BIRC2 transcription start site. ChIP-qPCR assay results indicated that siCITED4 significantly reduced the fold-enrichment of promoter-binding templates in PANC-1 cells (Fig. 4D). To confirm this, the *BIRC2* promoter assay was performed, and we found that siCITED4 decreased luciferase activity (Fig. 4E), indicating that CITED4 regulates *BIRC2* expression through transcriptional regulation.

Next, to examine the clinical relevance of CITED4 and BIRC2, we analyzed their expression levels in PC patient tissues. Our results showed that *CITED4* and *BIRC2* mRNA levels were significantly elevated in tumor tissues compared to normal tissues (Fig. 4F). Moreover, we observed a positive correlation between *CITED4* and *BIRC2* expression (Fig. 4G). To further validate these findings, we analyzed *CITED4* and *BIRC2* expression using GEPIA2 and GEO datasets, which also demonstrated a consistent positive correlation (Fig. 4H and Supplementary Fig. 3 A). Additionally, survival analysis using PAAD-TCGA data through Q-omics software revealed that patients with high *CITED4* and *BIRC2* expression had significantly poorer survival outcomes compared to those with low expression levels, but no difference was found upon considering only *BIRC2* levels (Supplementary Fig. 3 B, C).

To identify the downstream signaling pathways regulated by BIRC2, we performed RNA sequencing analysis after BIRC2 knockdown in PANC-1 and L3.6pl-GR cells. Upon BIRC2 silencing, 33 genes were significantly downregulated in both cell lines (Fig. 4I). KEGG pathway analysis revealed enrichment in cancer-related and apoptosis-related pathways (Fig. 4J), while GSEA analysis further identified associations with apoptosis and cell cycle regulation (Supplementary Fig. 3 D). Furthermore, we found that silencing BIRC2 or CITED4 led to the activation of p38 and JNK, key regulators of apoptosis within the MAPK signaling pathway (Fig. 4K and Supplementary Fig. 3E). This finding, along with the RNA sequencing results, suggests that BIRC2 regulates apoptosis through multiple signaling pathways. Collectively, these findings suggest that CITED4 directly regulates BIRC2 transcription, and BIRC2 expression promotes downstream signaling that enhances cell proliferation and inhibits apoptosis, ultimately contributing to the GEM-resistant phenotype in PC.

### Knockdown of *BIRC2* induces cellular apoptosis in GEM-resistant PC cells

**Fig. 5:**
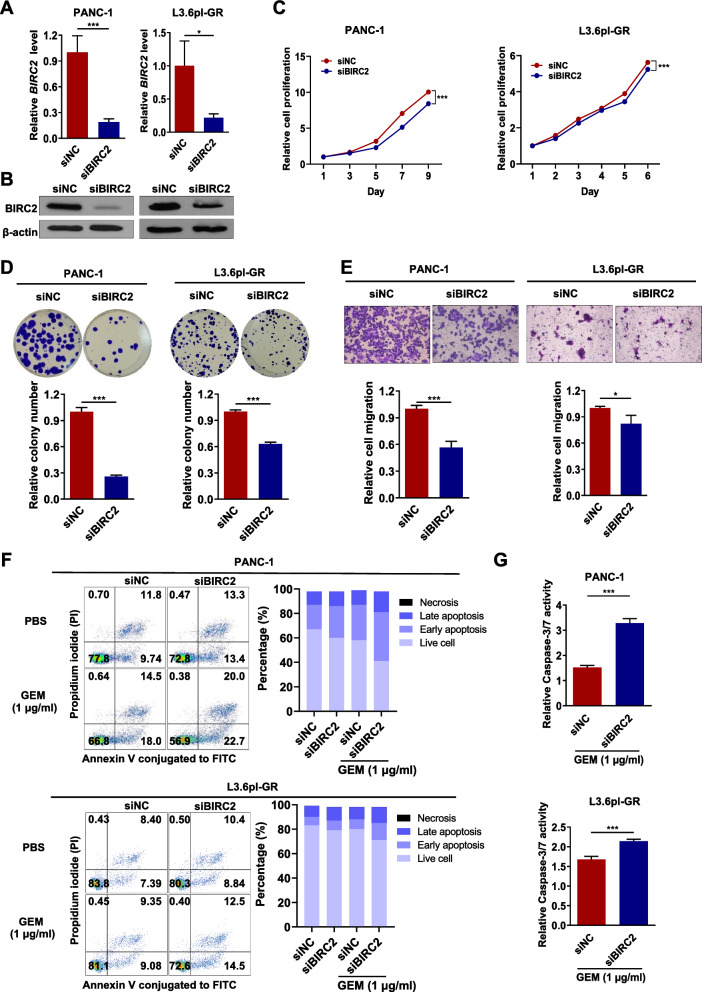
BIRC2 mediates GEM-resistance features related to CITED4 in PC.** A** mRNA and **B** protein expression levels of BIRC2 in *BIRC2*-knockdown PANC-1 and L3.6pl-GR cells. β-Actin was used as an internal control for mRNA and protein expression. **C** Cell viability was measured using a CCK-8 assay. **D** Representative image of the colony-formation assay in PANC-1 and L3.6pl-GR (top) cells. Relative quantification of the colony number (bottom). **E** Representative image of the cell-migration assay (top). Relative quantification of the number of migrated cells (bottom). **F** Comparative analysis of apoptosis after GEM treatment of *BIRC2*-knockdown PANC-1 and L3.6pl-GR cells using flow cytometry. Fluorescence-activated cell sorting analysis of siNC- and siBIRC2-transfected cells treated with (1 µg/ml) or without GEM for 48 h, following which the cells were stained with Annexin V-FITC and propidium iodide (upper panel). Percentages of viable, early apoptotic, late apoptotic, and necrotic cells. **G** Caspase-3/7 activity was measured using Caspase-Glo 3/7 Assay kit. *BIRC2*-knockdown PANC-1 (upper) and L3.6pl-GR (bottom) cells were treated with 1 µg/ml GEM for 48 h. *P < 0.05 and ***P < 0.001

To understand the mechanism of action of BIRC2 in PC in detail, a *BIRC2*-knockdown experiment was performed. Treatment with siBIRC2 reduced the mRNA and protein levels in PANC-1 and L3.6pl-GR cells (Fig. 5A, B). BIRC2 knockdown significantly reduced the cell proliferation, clonogenicity, and migration of PANC-1 and L3.6pl-GR cells (Fig. 5C–E).

To further assess the contribution of apoptosis by silencing of BIRC2, flow cytometry analysis was performed using PANC-1 and L3.6pl-GR cells. After treatment of PANC-1 and L3.6pl-GR cells with BIRC2 knockdown with 1 μg/ml GEM, an increase in cellular apoptosis rate was observed as compared with that in cells transfected with siNC (Fig. 5F). Moreover, the reduced BIRC2 expression resulted in increased Caspase3/7 activity in PANC-1 and L3.6pl-GR cells following GEM treatment (Fig. 5G). Collectively, these results were similar to those for siCITED4-treated PC cells, suggesting that the CITED4 downstream gene, BIRC2, plays a critical role in PC oncogenic properties and is associated with GEM resistance via regulation of apoptosis.

### Suppression of CITED4 increases GEM sensitivity in mice

To substantiate the role of CITED4 in GEM resistance PC in vivo, we established *CITED4*-KO cells using CRISPR-Cas9 in PANC-1 cells (Supplementary Fig. 4 A, B). *CITED4*-KO cells had a significantly decreased colony-forming ability as compared with normal PANC-1 cells under GEM treatment conditions in vitro (Supplementary Fig. 4 C). Moreover, *CITED4*-KO cells treated with GEM showed significantly induced cellular apoptosis as compared with PANC-1 cells, as confirmed using FACS analysis and Caspase3/7-Glo assay (Supplementary Fig. 4 D, E).

**Fig. 6:**
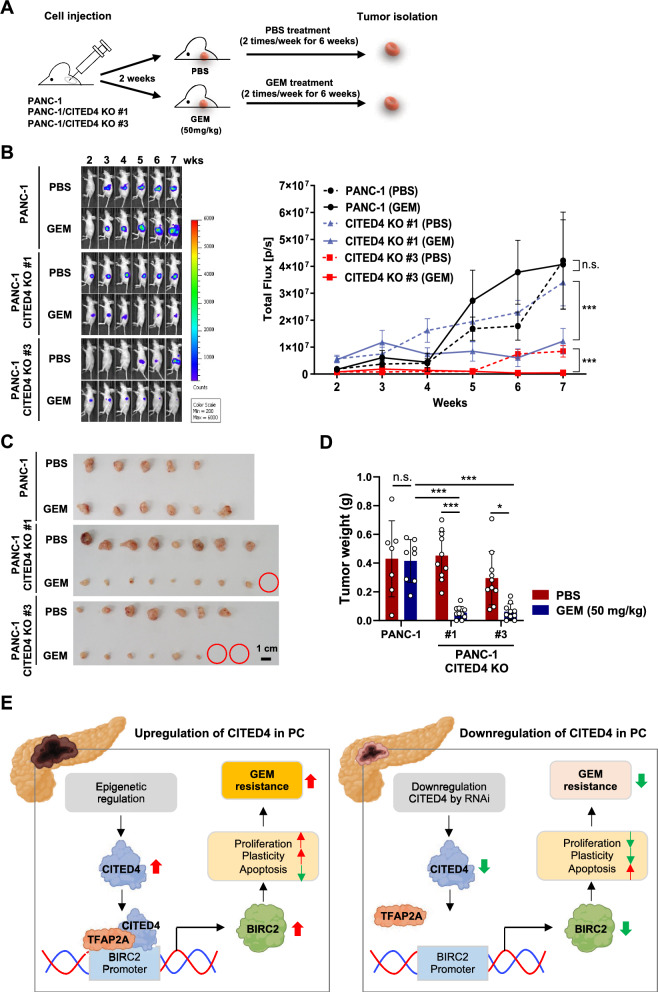
Knockout of *CITED4* decreases pancreatic tumor growth in an orthotopic mouse model.** A** Schematic of the experimental procedure. PANC-1 (PBS,* n* = 5; GEM, *n* = 6), *CITED4* knockout PANC-1 #1 (PBS, *n* = 8; GEM, *n* = 9) or #3 (PBS, *n* = 7; GEM, *n* = 8) cell were implanted to pancreas of BALB/c nude mice. After 2 weeks, the mice were treated with GEM (50 mg/kg) twice per week for 6 weeks. **B** Tumors were detected in mice using in vivo bioluminescence imaging (left), and bioluminescent intensities were quantified (right). Two-way ANOVA was used for statistical analysis. **C** Macroscopic images of each tumor (scale bar, 1 cm). Red circles indicate complete regression. **D** Average of tumor weight. **E** Schematic representation of the roles of CITED4 in GEM-resistant PC. *P < 0.05 and ***P < 0.001

Next, we established pancreatic orthotopic tumor models by implanting PANC-1 or *CITED4*-KO PANC-1 cells into the pancreas of BALB/c nude mice followed by intraperitoneal administration of PBS or GEM twice a week (Fig. 6A). The tumor size was quantitatively measured via luciferase activity using the IVIS200 imaging system. Remarkably, we observed a significantly reduced luciferase activity in both CITED4-KO-#1 and -#3 GEM-treated mice as compared with that in their PBS-treated counterparts (Fig. 6B and Supplementary Fig. 5 A), but there was no significant difference in body weight (Supplementary Fig. 5 B). Notably, luciferase activity was not different between PBS- and GEM-treated mice in the PANC-1 cell injection group. Similarly, the tumor weight significantly decreased in both groups (CITED4-KO-#1 and -#3) under GEM treatment as compared with that in the PBS-treated group but not in the groups injected with parental PANC-1 cells (Fig. 6C, D). Taken together, these findings provide compelling evidence that CITED4 mediates GEM-resistant PC features, in part, by directly activating the expression of BIRC2, encoded by a CITED4-target gene, with known anti-apoptotic functions, both in vitro and in vivo.

## Discussion

PC is one of the most serious malignancies worldwide, and it has a low survival rate. Despite the recent use of immunotherapy for PC treatment, GEM remains the primary approach. However, the clinical utility of GEM is hampered by the development of resistance mechanisms. Long-term treatment of GEM often leads to acquired drug resistance, driven by the cancer stem cells, tumor microenvironment, epithelial-mesenchymal transition, and activation of various oncogenic pathways. Therefore, it is crucial to explore the underlying causes and mechanisms responsible for GEM resistance in PC.

First, we discovered elevated CITED4 expression in the GEM-resistant PC mouse model via RNA sequencing. Moreover, we observed a correlation between CITED4 expression and poorer survival outcomes in PC patients. Recent studies have sought to elucidate the role of CITED4 in various cancers including breast cancer, colorectal cancer, and lung adenocarcinoma [23, 25, 34]. CITED4 is predominantly located in the nucleus; however, cytoplasmic translocation or loss of nuclear expression of CITED4 has been observed in breast cancer development, where it might represent a prognostic marker [34]. In lung cancer, *CITED4* is induced by heparin-binding epidermal growth factor (HB-EGF) through signal transducer and activator of transcription (STAT3)-dependent pathway, resulting in cell proliferation. Additionally, a positive correlation was observed between *HB-EGF* and *CITED4* in primary lung tumors [22]. More recently, Zhang et al. reported that CITED4 enhances the metastatic potential of lung adenocarcinoma by interacting with CTNNB1, resulting in the upregulation of claudin 3 expression [25]. However, previous investigations did not explore CITED4-related mechanisms of drug resistance. Our study sheds light on the potential association between CITED4 expression and GEM resistance in PC. Notably, suppression of CITED4 in L3.6pl-GR and PANC-1 cells led to a significant reduction in their oncogenic properties, with a particular impact on cellular apoptosis. Additionally, in vivo experiments revealed that *CITED4*-KO PANC-1 tumors increased the sensitivity to GEM, indicating that CITED4 regulates GEM resistance in PC by modulating cellular apoptosis.

Previously, it has been reported that GEM has the ability to reactivate epigenetically silenced genes and acts as DNA methyltransferase inhibitor [35]. These findings revealed that GEM inhibited DNA methyltransferase activity, leading to the destabilization of the DNMT1 protein. Consequently, several epigenetically silenced genes including GSTP1, IGFBP3, and RASSF1 A were reactivated. In another study, it was noted that CITED4 expression is regulated by DNA methylation, and its expression exhibits an inverse correlation with DNA methylation status [24]. Our results also showed that CITED4 can be regulated by DNA methylation and/or histone modifications, suggesting that CITED4 is induced by destabilization of DNMT1 via the GEM-resistant status, thus the reactivated CITED4 is directly associated with the GEM-resistance in PC. Additionally, our findings identified CpG site hypomethylation in the CITED4 promoter region as a key factor contributing to CITED4 upregulation in GEM-resistant conditions. This was further supported by public data analysis, which revealed an inverse correlation between CITED4 expression and DNA methylation status, providing more direct evidence that CITED4 expression can be regulated through DNA methylation mechanisms.

In the present study, to identify the downstream target of CITED4, we first evaluated apoptosis-related genes using an apoptosis array, because the apoptosis genes have been closely linked to drug resistance [36–38]. We identified the Baculoviral IAP Repeat Containing 2 (BIRC2), which was regulated depending on the CITED4 expression level. BIRC2 has been extensively studied in cancer cells and is recognized as a negative regulator of cellular apoptosis. Additionally, BIRC2 is involved in activating MAPK signaling and its downstream effector molecules contribute to various oncogenic processes, including proliferation, mitosis, cell survival, and apoptosis [39–42]. Previous studies have demonstrated the role of BIRC2 in promoting tumorigenesis and inhibiting apoptosis in several cancer types, including gallbladder cancer and ovarian cancer [40–44]. Depletion of BIRC2 has been shown to enhance chemosensitivity in ovarian cancer [44, 45], highlighting its potential as a therapeutic target. Moreover, IAP antagonists, such as AZD5585 and HM822, induce apoptosis in PC by targeting XIAP and BIRC2 [46–50]. Our findings also showed that BIRC2 plays a crucial role in cellular apoptosis as well as cell growth and motility, suggesting that CITED4-mediated BIRC2 expression promotes cancer cell malignancies by inhibiting the apoptotic pathway and activating oncogenic properties; thus, the CITED4-BIRC2 axis represents a potential regulator of drug resistance in PC.

This study highlights cell cycle and apoptosis signaling pathways as key mechanisms within the CITED4-BIRC2 axis, which enhances cancer cell survival in GEM-resistant PC. Among various GEM resistance mechanisms, we identified these pathways as crucial in promoting tumor cell survival. Specifically, RNA sequencing analysis of BIRC2 knockdown cells revealed a set of genes that were significantly downregulated compared to controls, including NRAS proto-oncogene, GTPase (NRAS), recombination signal binding protein for immunoglobulin kappa J region (RBPJ), ubiquitin conjugating enzyme E2 N (UBE2 N), ZW10 interacting kinetochore protein (ZWINT), and MDM2 proto-oncogene (MDM2), which have been previously reported to be involved in drug resistance. These genes play essential roles in various cancers, contributing to cell proliferation, apoptosis inhibition, and tumor malignancy [51–54]. RBPJ has been shown to interact with TRIM59, activating the Notch signaling pathway, which promotes GEM resistance in PC [51]. UBE2 N was identified as a binding partner of TRIM11 through co-immunoprecipitation analysis, and its upregulation in PC suggests its role in TAX1BP1 signaling, contributing to GEM resistance [52]. ZWINT is known to regulate p53, promoting its ubiquitination and degradation, thereby enhancing PC cell proliferation [53]. MDM2 is a well-known regulator that reduces p53 protein stability, and treatment with an MDM2 inhibitor stabilizes p53, leading to growth inhibition, apoptosis induction, and increased GEM sensitivity in PC cells [54]. Additionally, we performed a correlation analysis between these genes and *BIRC2* expression in the PAAD-TCGA dataset using GEPIA2, which revealed a positive correlation between *BIRC2* expression and *NRAS*, *RBPJ*, *UBE2 N*, *ZWINT*, and *MDM2* (Supplementary Fig. 6 A). Furthermore, we found that higher *ZWINT* expression was associated with lower survival in PC patients, and those with simultaneously elevated expression of *ZWINT* and *CITED4* or *ZWINT* and *BIRC2* exhibited even poorer survival outcomes (Supplementary Fig. 6B). Collectively, these findings suggest that CITED4-mediated BIRC2 expression ultimately contributes to GEM resistance by regulating the drug resistance-related genes, thereby influencing anti-apoptosis and cell cycle pathways, ultimately promoting chemoresistance in PC.

However, this study has a limitation in that it does not fully elucidate the mechanisms underlying CITED4 upregulation in GEM-resistant PC. To address this, further research is required to investigate the complex regulatory networks between CITED4 expression and its upstream signaling pathways, such as those involving inflammatory signaling, epigenetic alteration, or microRNAs. Additionally, other CITED4 downstream targets can also contribute to CITED4-mediated GEM resistance in PC, although no experimental evidence supporting this was presented in this study, since the apoptosis array demonstrated decreased expression of Survivin, HIF1a, and Claspin following *CITED4* silencing. Furthermore, while PANC-1 and L3.6pl-GR were used as GEM-resistant PC cell models, the CITED4-mediated resistance mechanism identified in this study may not be fully generalizable to all PC patients. Therefore, further studies utilizing a broader range of GEM-resistant cell lines are necessary to gain a more comprehensive understanding of resistance mechanisms. Similarly, although the orthotopic animal model used in this study provides a physiologically relevant system, it does not fully replicate the clinical environment. To strengthen the clinical relevance of our findings, additional research using various PC animal models and clinically relevant systems is needed to further explore the relationship between CITED4 and GEM resistance. Taken together, to fully understand the CITED4-mediated signaling networks and evaluate the clinical significance of these findings, further studies are needed to elucidate the biological role of signaling molecules in GEM resistance in PC.

## Conclusion

In this study, we found elevated CITED4 expression in GEM-resistant PC cells and surveyed the putative downstream genes that may suppress apoptotic signaling pathways to maintain chemoresistance. Using an apoptosis array, we identified an important downstream gene, *BIRC2,* that suppresses apoptosis. RNAi against CITED4 decreased *BIRC2* expression levels in GEM-resistant PC cells, suggesting that *BIRC2* expression is regulated by CITED4. Further validation using ChIP and promoter assays confirmed a direct regulatory interaction between CITED4 and BIRC2 in PC cells. Additionally, analysis of PC patient tissues and the PAAD-TCGA cohort revealed a positive correlation between CITED4 and BIRC2 expression levels, further supporting their functional link. In vivo experiments showed that *CITED4* KO increased GEM sensitivity in PANC-1 orthotopic mice, indicating that CITED4 could play an important role in GEM resistance in PC. The proposed mechanisms are illustrated in Fig. 6E. To translate these findings into clinical applications, future studies can focus on validating CITED4 and BIRC2 as specific biomarkers for GEM-resistant PC patients. Moreover, further research is needed to develop CITED4-specific inhibitors to enhance GEM sensitivity, potentially leading to more effective combination treatment strategies for PC. Taken together, our findings support the concept that targeting the CITED4–BIRC2 axis could be a rational approach to enhance the survival of patients with GEM-resistant PC.

## Supplementary Information

Additional file 1.

## Data Availability

The datasets used and/or analyzed in the current study are available from the corresponding author upon reasonable request.

## Abbreviations

BIRC2: Baculoviral IAP repeat containing 2
CITED4: Cbp/p300-interacting transactivator 4
GEM: Gemcitabine
KO: Knockout
PC: Pancreatic cancer
KO: Knockout
GSEA: Gene Set Enrichment Analysis
TCGA: The Cancer Genome Atlas
PAAD: Pancreatic adenocarcinoma
TSA: Trichostatin A
JNK: C-Jun N-terminal kinase
MAPK: Mitogen-activated protein kinase
HB-EGF: Heparin-binding epidermal growth factor
STAT3: Signal transducer and activator of transcription
XIAP: X-linked inhibitor of apoptosis
NRAS: NRAS proto-oncogene, GTPase
RBPJ: Recombination signal binding protein for immunoglobulin kappa J region
UBE2 N: Ubiquitin conjugating enzyme E2 N
ZWINT: ZW10 interacting kinetochore protein
MDM2: MDM2 proto-oncogene
