# Supplementary material for: Gemcitabine resistance by CITED4 upregulation via the regulation of BIRC2 expression in pancreatic cancer
Source: J Biomed Sci. 2025 May 19;32:49. doi: 10.1186/s12929-025-01140-y (PMC12090687; doi:10.1186/s12929-025-01140-y)
Supplement: Supplementary file 1 — Additional file 1. [file 12929_2025_1140_MOESM1_ESM.docx]

**Supplementary Information**

**Table S1. List of primers**

| **Gene** | **Primer (Forward)** | **Primer (Reverse)** | |
| --- | --- | --- | --- |
| qPCR | | | |
| CITED4 | CTCCTGCGGGAGGACAGTTT | | CAATGGCTCAGATTGGGGGA |
| BIRC2 | CAGACACATGCAGCTCGAATGAG | | CACCTCAAGCCACCATCACAAC |
| TFAP2A | ATACGGATAGTCCCCGTTCC | | GATGAACCTCCGGGAAAGAC |
| TFAP2A-var3 | ATCGTGCGTCAGAGTGAGC | | CGCCGACAAGGAGATACG |
| GAPDH | CTCTGCTCCTCCTGTTCGAC | | TTAAAAGCAGCCCTGGTGAC |
| ACTB | TCCTCTCCCAAGTCCACACAGG | | GGGCACGAAGGCTCATCATTC |
| promoter assay | | | |
| TFAP2A | AAGCTAGCATACGGATAGTCCCCGTTCCCCT | TTAAGCTTGATGAACCTCCGGGAAAGACGCTG | |
| TFAP2A-var3 | AAGCTAGCATCGTGCGTCAGAGTGAGCCCG | TTAAGCTTCGCCGACAAGGAGATACGGCCCG | |
| cloning | | | |
| CITED4-cas9 | CACCGGACGCCGAACTCATCGACG | AAACCGTCGATGAGTTCGGCGTCC | |

**Table S2. siRNA sequences**

| **Gene** | **sense** | **antisense** |
| --- | --- | --- |
| siCITED4 | CAGCUUUCGGACUCUGGUU | AACCAGAGUCCGAAAGCUG |
| siBIRC2 | CUCAGUAACUGGGAACCAA | UUGGUUCCCAGUUACUGAG |

**Table S3. List of antibodies**

| **Antibodies** | **Company** | **Catalog No.** | **Dilution** |
| --- | --- | --- | --- |
| Primary antibodies | | | |
| anti-CITED4 | Abcam | ab 134072 | 1:100 (for IHC)  1:1,000 (for WB) |
| anti-CITED4 | Thermo fisher | MA-16077 | 1:50 (for IF) |
| anti-BIRC2 | Abcam | ab108361 | 1:100 (for IHC, IF)  1:1,000 (for WB) |
| anti-cleaved Caspase-3 | Cell signaling | #9662 | 1:1,000 (for WB) |
| anti-α-tubulin | Sigma | T5168 | 1:1,000 (for WB) |
| anti- Phospho-JNK | Cell signaling | #4668 | 1:1,000 (for WB) |
| anti-JNK | Cell signaling | #9252 | 1:1,000 (for WB) |
| anti-Phospho-p38 | Cell signaling | #4511 | 1:1,000 (for WB) |
| aanti-p38 | Cell signaling | 8690 | 1:1,000 (for WB) |
| GAPDH | Satacux | Sc-32233 | 1:1,000 (for WB) |
| anti-ACTB | AbClon | AbC-2004 | 1:1,000 (for WB) |
| ProLong^TM^ Gold antifade reagent with DAPI | Thermo fisher | P36931 |  |
| Secondary antibodies | | | |
| anti-Mouse- IgG-HRP | Cell signaling | #7076 | 1:1,000 (for WB) |
| anti-Rabbit-IgG-HRP | Cell signaling | #7074 | 1:1,000 (for WB) |
| anti-Goat-Alexa488 | Thermo fisher | a-11029 | 1:200 (for IHC) |
| anti-Rabbli-Alexa488 | Cell signaling | #4412 | 1:100 (for IF) |
| Alexa-Fluor 594 Phalloidin | Thermo fisher | A12381 |  |

**Table S4. STR analysis results of parental cells and primary cultured cells from orthotopically implanted pancreatic tumor.**

| Sample | L3.6pl (parental cell) | Ctrl-L3.6pl | GE-L3.6pl |
| --- | --- | --- | --- |
| D8S1179 | 14 | 14 | 14 |
| D21S11 | 29, 32.2 | 29, 32.2 | 29, 32.2 |
| D7S820 | 8, 10 | 8, 10 | 8 |
| CSF1PO | 11, 12 | 11, 12 | 11, 12 |
| D3S1358 | 15 | 15 | 15 |
| TH01 | 7 | 7 | 7 |
| D13S317 | 8 | 8 | 8 |
| D16S539 | 9, 12 | 9 | 9 |
| D2S1338 | 24 | 24 | 24 |
| D19S433 | 11, 13 | 11, 13 | 11, 13 |
| Vwa | 18 | 18 | 18 |
| TPOX | 8 | 8 | 8 |
| D18S51 | 13 | 13 | 13 |
| Amelogenin | X | X | X |
| D5S818 | 11 | 11 | 11 |
| FGA | 25, 27 | 25 | 25, 27 |

**Supplementary Fig. 1 Analysis of viability and caspase 3/7 activity in L3.6pl-GR cells.** **A** Cell viability was measured using the Cell Titer-Glo kit. Control and L3.6pl-GR cells were incubated with 0.01 μg/mL gemcitabine (GEM) for 48 h. Data represent the mean ± standard deviation (SD) and were analyzed using Student’s *t*-test (^***^*P* < 0.001). **B** Caspase-3/7 activities were measured using the Caspase-Glo 3/7 Assay kit. Control and L3.6pl-GR cells were treated with 1 μg/ml GEM for 48 h. Data represent the mean ± SD and were analyzed using Student’s *t*-test (^*^*P* < 0.05 and ^***^*P* < 0.001).


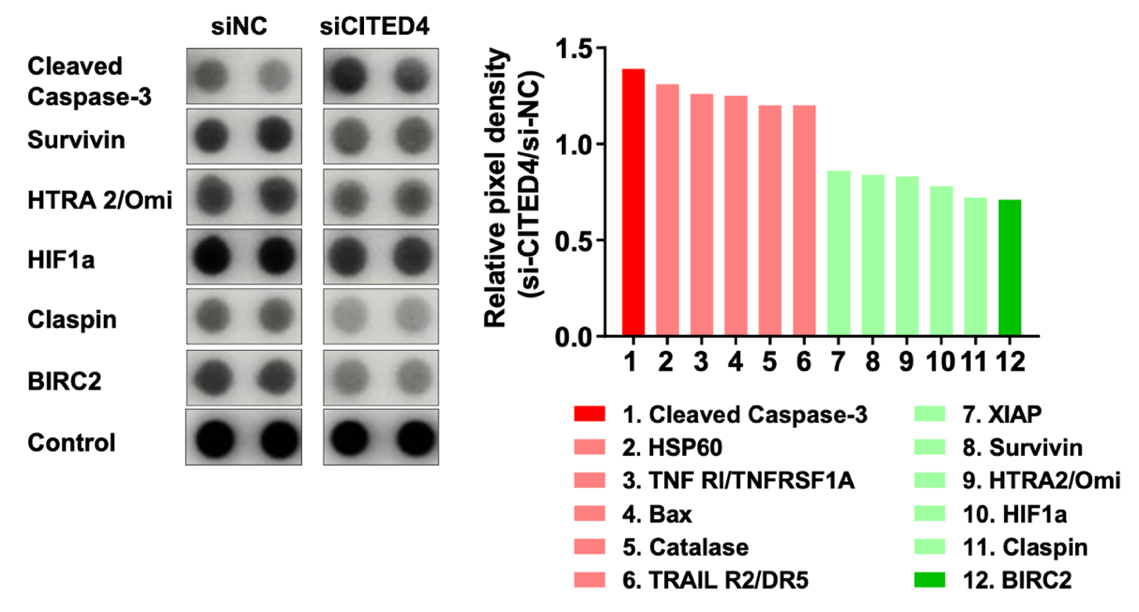


**Supplementary Fig. 2 Comparison of apoptosis-related protein expression between siCITED4- and siNC-transfected L3.6pl-GR cells.** Spot densities of apoptosis-related proteins were analyzed using ImageJ. The expression of cleaved caspase-3 was upregulated whereas that of survivin, HTRA 2/Omi, HIF1a, claspin, and BIRC2 was downregulated following treatment with siCITED4.


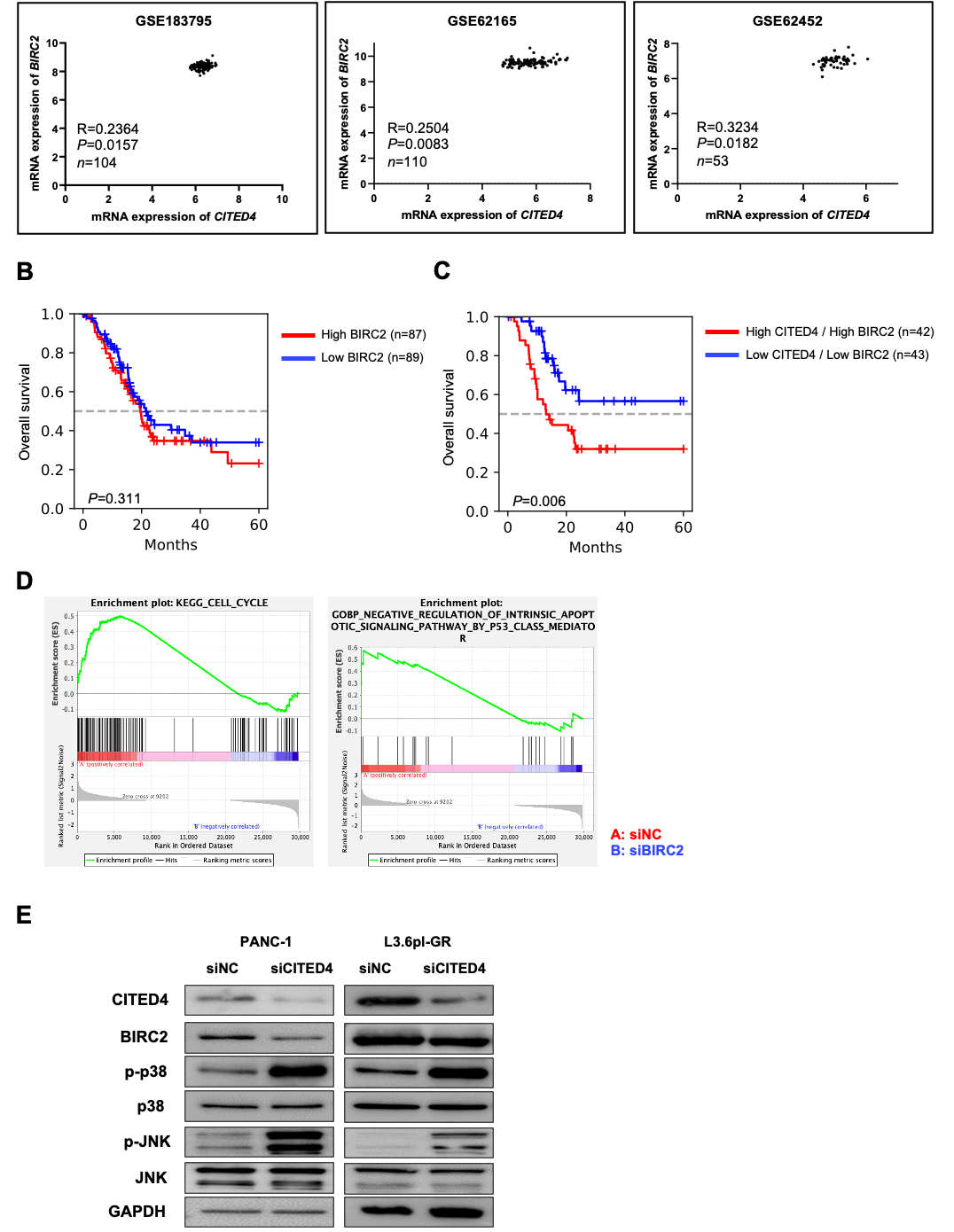


**Supplementary Fig. 3 BIRC2 downstream signaling is associated with cell cycle regulation and apoptosis. A** Correlation analysis of *CITED4* and *BIRC2* expression levels using GEO dataset (GSE183795, GSE62165 and GSE62452). **B** Overall survival analysis for PC samples from Q-omics (https://qomics.sookmyung.ac.kr/) for *BIRC2*. **C** Overall survival analysis for the combination of *CITED4* and *BIRC2*. Statistical significance between groups was assessed using the log-rank test. **D** Gene Set Enrichment Analysis (GSEA) analysis of 33 differentially expressed genes (DEGs) associated with the Cell Cycle pathway (KEGG analysis, left panel) and Apoptosis pathway (GO analysis, right panel). **E** Protein expression changes were analyzed to assess the impact of CITED4 silencing on apoptotic pathways, with GAPDH serving as an internal control.


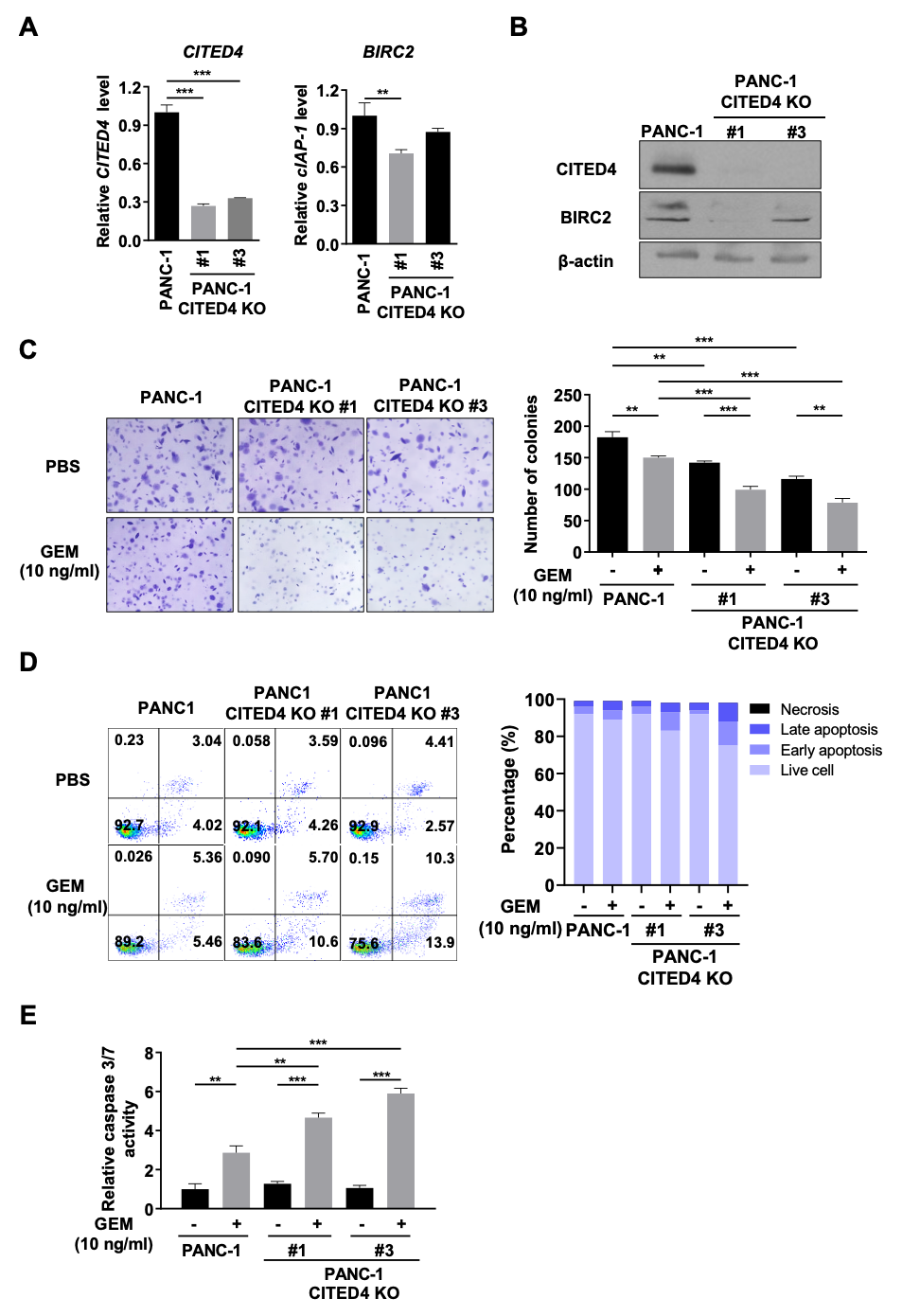


**Supplementary Fig. 4 Establishment of the *CITED4*-knockout (KO) PANC-1 cell line. A** mRNA expression levels of *CITED4* (left panel) and *BIRC2* (right panel) in CITED4-KO PANC-1cells, as revealed using the CRISPR-Cas9 system. Actin was used as an internal control for mRNA expression analysis. **B** Protein expression levels of CITED4 and BIRC2 in *CITED4*-KO PANC-1 cells. **C** Representative images (left panel) and quantification of colony number (right panel) for the colony-formation assay in PANC-1 and *CITED4*-KO PANC-1 cells with or without GEM treatment. **D** Comparative analysis of apoptosis following treatment of *CITED4*-KO PANC-1 cells with GEM using flow cytometry. Fluorescence-activated cell sorting analysis of siNC- and siCITED4-transfected cells, treated with (10 ng/ml) or without GEM for 48 h and then stained with Annexin V-FITC and propidium iodide (PI; upper panel). Percentage of viable (white bars), early apoptotic (light gray bars), late apoptotic (dark gray bars), and necrotic (black bar; bottom panel) cells. **E** Caspase-3/7 activities were measured using the Caspase-Glo 3/7 assay kit. *CITED4*-KO #1 and #3 PANC-1 cells were treated with 10 ng/ml GEM for 48 h. Data represent the mean ± SD (PANC-1 and PANC-1-CITED4-CAS9-#1 and -#3) and were analyzed using one-way ANOVA (^***^*P* < 0.001).

**Supplementary Fig. 5 Knockout of *CITED4* decreases pancreatic cancer tumor growth in an orthotopic mouse model. A** *In vivo* bioluminescence images of all mice. **B** Mouse body weight was measured every week for 7 weeks.

**
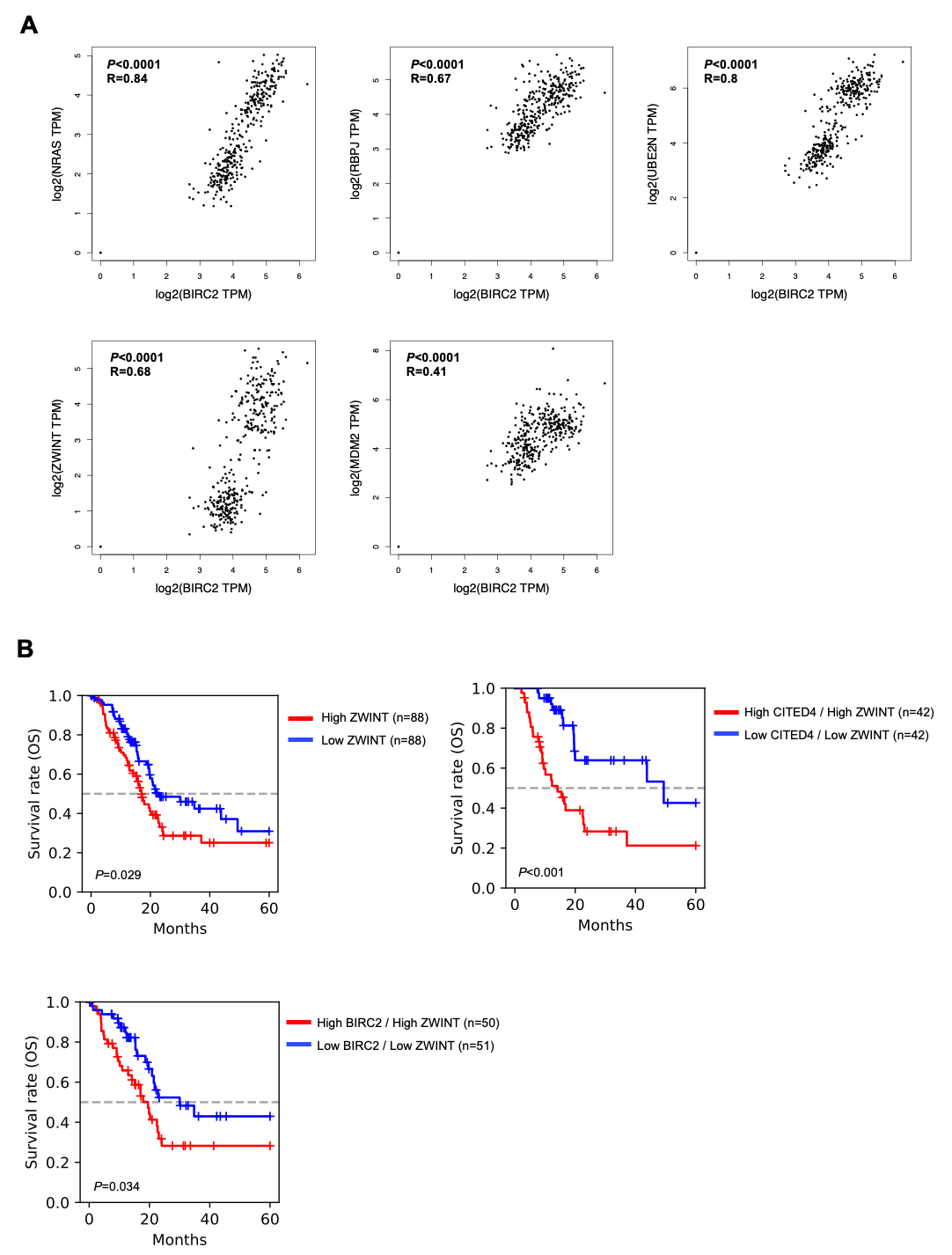
**

**Supplementary Fig. 6 Correlation and survival analysis of between BIRC2 and its downstream genes. A** Correlation analysis between *BIRC2* and its downstream genes, including *NRAS*, *RBPJ*, *UBE2N*, *ZWINT* and *MDM2*. **B** Overall survival analysis of PC samples from Q-omics (https://qomics.sookmyung.ac.kr/) for ZWINT, as well as the combined expression of CITED4 and ZWINT or BIRC2 and ZWINT. Statistical significance between groups was determined using the log-rank test.
